# Usage of Virtual Reality Technology in Physiotherapy in Germany: Results from a Survey

**DOI:** 10.3390/bioengineering12020106

**Published:** 2025-01-23

**Authors:** Alexander Elser, Maybritt Ohse, Camilla Frankenstein, Madlin Leeuw, Sophie Schiebler, Sandra Schmieder, Syl Slatman, Axel Georg Meender Schäfer

**Affiliations:** 1 Faculty of Social Work and Health, University of Applied Sciences and Arts Hildesheim/Holzminden/Göttingen, 31134 Hildesheim, Germany; maybritt.ohse1@stud.hawk.de (M.O.); camilla.frankenstein@stud.hawk.de (C.F.); madlin.leeuw@stud.hawk.de (M.L.); sophie.schiebler1@stud.hawk.de (S.S.); sandra.schmieder1@stud.hawk.de (S.S.); axel.schaefer@hawk.de (A.G.M.S.); 2 Department Therapy Science I, Faculty 4 for Human Sciences, Brandenburg University of Technology Cottbus-Senftenberg, 03025 Cottbus, Germany; 3 Department of Musculoskeletal Rehabilitation Research Group, HAN University of Applied Sciences, 6503 Nijmegen, The Netherlands; syl.slatman@han.nl

**Keywords:** virtual reality, VR, physiotherapy, survey, usage, barriers

## Abstract

With an aging population and an increasing prevalence of non-communicable diseases, Germany’s healthcare system is facing significant challenges that require innovative solutions such as digitalization. Among digital technologies, virtual reality (VR) has shown promise in various healthcare settings; however, its use in physiotherapy practice is unknown. This study aimed to assess the frequency and use of therapeutic VR among physiotherapists in Germany and to identify barriers to its adoption. A cross-sectional survey of 296 physiotherapists was conducted, with responses indicating that only 2.7% had used therapeutic VR in the past year. Most physiotherapists were unfamiliar with VR therapy, suggesting that lack of awareness is the primary barrier. Despite limited current use, a significant proportion of physiotherapists were open to integrating VR technologies in the future. Our findings highlight the need for increased information about therapeutic VR within the physiotherapy community and suggest potential growth as awareness and institutional support increases. Future strategies should focus on promoting the benefits of VR and integrating it into reimbursement frameworks to facilitate wider adoption in patient care.

## 1. Introduction

With an aging global population and an increasing burden of non-communicable diseases such as musculoskeletal disorders, diabetes and stroke, healthcare systems are facing significant challenges [1]. In 2022, more than a fifth of the German population was aged 65 or older. According to demographic projections, this percentage will rise to 28% by 2050 [2]. This will also lead to a rising number of people affected by musculoskeletal diseases and resulting chronic impairments in Germany [1,3]. The German healthcare system is already experiencing funding problems and costs in the healthcare professions have risen sharply in recent years [4]. A closer look at the costs spent on physiotherapy reveals a high demand for outpatient first care physiotherapy. In 2021, there were over 34 million medical prescriptions distributed among approximately 8.7 million members of a public health insurance company [4]. Expenditure on prescribed outpatient therapies reported by this insurance company totaled more than EUR 1.4 billion in 2021. Physiotherapy accounted for 73.1% of the costs incurred in this area, with an increase of at least 50% over the last 5 years [4]. This highlights the need for innovative solutions to ensure adequate healthcare in the future.

Digitalization offers an opportunity for more effective and therefore more cost-efficient care in the healthcare system in general and in the therapy professions in particular. To ensure an equitable and appropriate medical service, the German government has responded with the policy Accelerating the Digitalization of the Healthcare System [5], which aims to support digital technologies to meet the current challenges in the healthcare system. One of these digital technologies is virtual reality (VR). Positive effects of VR interventions have already been observed in, for example, the treatment of patients undergoing chemotherapy [6], after stroke [7,8], after spinal cord injury [9], and also in elderly people with cognitive impairment [10] and in children undergoing clinical procedures [11].

When implementing VR interventions in healthcare, there is already initial knowledge about the barriers and facilitators that may be encountered. Previous research has identified organizational structures and the VR technology itself as barriers to implementing VR interventions in various healthcare settings [12,13,14]. Regarding the use of VR in physiotherapy, the VR device itself seems to be a major barrier due to technical limitations and inappropriate VR devices for specific patients and their needs. Also, a lack of appropriate tutorials in the VR software and lack of protocols for VR interventions are barriers, and patient-related factors such as low gaming skills play a role in the implementation of VR interventions [15,16]. Conversely, staff and healthcare professionals can act as facilitators by reducing anxiety about new technologies and changing patients’ attitudes towards VR. In particular, data security and potential dependencies arising from the use of VR in healthcare [17] can cause uncertainty for both patients and therapists. Healthcare professionals are also generally interested in using VR in rehabilitation and have positive expectations of therapeutic VR in treatment and rehabilitation. In addition, patients also have positive expectations, describing VR as a treatment that they find fun and enjoyable [13,14,15,16].

There is still very little research into whether and how therapeutic VR is being used in healthcare and physiotherapy. Virtual reality has been widely used in medicine during the COVID-19 pandemic, with positive effects on the treatment of various health conditions [18]. A survey in the Netherlands showed that only about 7% of physiotherapists there use therapeutic VR. A regression analysis showed that only the size of the practice was associated with the use of VR [19]. However, the frequency of use in physiotherapy in Germany is currently unknown.

Therefore, the aim of this study was to describe how many physiotherapists in Germany use therapeutic VR. Secondary aims were to describe how they use therapeutic VR and if they do not use it, what the reasons are.

## 2. Materials and Methods

A cross-sectional survey was conducted to determine the extent of virtual reality intervention in physiotherapy in Germany. The survey did not require an approved ethics proposal as no vulnerable group was targeted and all data were collected and analyzed anonymously at all times. The research group is committed to respecting the German Research Foundation’s Guidelines for Safeguarding Good Scientific Practice [20] and the Declaration of Helsinki [21] throughout the research process. The data were collected in May and June 2024 using a SoSci Survey [22]. This study is reported in accordance with the Checklist for Reporting Results of Internet E-Survey (CHERRIES) [23].

### 2.1. Questionnaire

The questionnaire is based on a Dutch questionnaire used by Slatman et al., 2024 [19]. Items were adapted to reflect the differences between the Dutch and German health systems and to broaden the questionnaire from chronic pain treatment with therapeutic VR to general use in physiotherapy. For example, the item on the highest professional degree was adapted as there are different educational paths in physiotherapy in Germany than in the Netherlands. In order to cover the breadth of physiotherapy, an item was added asking about the area of application of therapeutic VR. The survey included five demographic questions (gender, age, practice size, years of experience as a physiotherapist, physiotherapy specialization). Physiotherapists who had used therapeutic VR in the past year had to answer 13 questions and physiotherapists who had not used therapeutic VR had to answer 7 questions. A filter question was used to guide participants to the relevant questions. The order of the questions was constant, with no alternation or randomization. To complete the survey, all questions had to be answered, and there was a ‘no information’ option in all questions if a physiotherapist did not wish to answer a particular question. The survey took physiotherapists approximately five minutes to complete if they used therapeutic VR and three minutes if they did not and there was no option to go back through the survey to change answers on previous pages. The survey was pilot tested by 8 physiotherapists who were not part of the research group and the survey can be found in the Appendix A.

### 2.2. Recruitment

The survey was open, with no password protection, as the aim of the recruitment strategy was to reach a wide range of diverse physiotherapists. Participants should preferably come from different areas of physiotherapy and have different experiences, qualifications or specializations. It was therefore decided to contact a national professional association, professional training centers, rehabilitation clinics, general hospitals and registered outpatient practices in urban or rural areas directly by email. In addition, personal contacts and social media, particularly Instagram, were used by the research group to disseminate the survey to potential participants. All participants responded to the survey voluntarily and received no compensation for their participation.

### 2.3. Sample Size

According to Mossig (2012) [24], the sample size must be at least 384 from a population of approximately 250,000 physiotherapists in Germany to deviate by no more than 5 percentage points from the actual result of the population with a confidence level of 95%.

### 2.4. Analysis

The collected data were downloaded and analyzed using SPSS version 28 (IBM corporation, Armonk, NY, USA). The demographic data were tested for normal distribution and presented by median, interquartile range (IQ) and frequencies. The results to the closed-ended questions were analyzed by calculating percentages and presented in tables and graphs. The results were then compared with a recent study using the same methods and aims in the Netherlands [19].

## 3. Results

Approximately 1000 emails were sent with a request for voluntary participation and an online link. Two reminder emails were sent one and four weeks later. In total, the survey was clicked on 1143 times. Of these, 266 physiotherapists completed the survey and 296 completed the survey to the extent that we considered it valid. This was the point at which participants voted on the primary research question of whether they had used therapeutic VR in the last 12 months. Therefore, 296 records were included in the analysis, the demographic characteristics of which are shown in Table 1. The median age of the total sample was 46 years with an IQ of 25 years. The majority of participants were female (66.2%) and held a vocational qualification as their highest level of qualification (68.2%). Nearly half of participants had a working experience of 20 years or more (49.7%).

Of the 296 participating physiotherapists, eight (2.7%) used therapeutic VR in their treatments in the past 12 months. The median age of these physiotherapists was 47 years (IQ 36). Compared to the physiotherapists who did not use VR, it appears that more of them had an academic degree and that there were either more than twenty other physiotherapists or only one physiotherapist employed in the facilities where they worked. There is no recognizable difference in terms of work experience.

Physiotherapists used therapeutic VR with patients of all ages. It was most commonly used for neurological conditions, chronic pain and musculoskeletal conditions. Almost all physiotherapists used therapeutic VR to activate their patients, but also other proposed working mechanisms such as relaxation and education are used. Full details of use are given in Table 2.

Seventeen of the total sample of physiotherapists used therapeutic VR in the past but no longer do so. The main reasons for stopping therapeutic VR were costs and dissatisfying results. Most of them used it for musculoskeletal conditions and are considering using therapeutic VR again in the future. See Table 3 for more details.

The main reason why physiotherapists in Germany had never used therapeutic VR was because they had never heard of it, with only a few stating that costs were the main barrier. Overall, 34.4% of all physiotherapists can imagine using therapeutic VR in the future, 41.4% said maybe and 18.3% said they will not use it in the future. Table 4 provides more information about how physiotherapists imagine using therapeutic VR in the future.

Compared to a study by Slatman et al. (2024) [19] in the Netherlands, the frequency of use of therapeutic VR in Germany is slightly different (Table 5). In Germany, 2.7% of physiotherapists use therapeutic VR, compared to 7.3% in the Netherlands. Adding the two samples together results in a frequency of use of 5.0% with a sample size of 515 physiotherapists.

## 4. Discussion

This was the first survey to gain insight into the use of therapeutic VR in physiotherapy care in Germany. Out of 296 physiotherapists, a minority of 2.7% had used therapeutic VR in the last twelve months. The main reason for this lack of use is that 67.2% of participants have never heard of therapeutic VR. However, 75.8% can imagine using therapeutic VR in their treatments in the future.

A comparison of the sample in this study with the population revealed slight differences. In terms of gender distribution, 74.6% of physiotherapists in Germany are female in the population [25]. In our sample, 66.2% of respondents classified themselves as female. In addition, 32.8% of the sample have an academic degree, which is significantly higher than in the population, as the scientific service of the German Bundestag stated a rate of 2.65% for physiotherapists with an academic degree in 2021 [26]. The distribution of physiotherapists in the different settings of outpatient care, clinical and rehabilitation centers reflects the population well [27].

The limited use of therapeutic VR is also reflected in a recent survey in the Netherlands. There, 7% of physiotherapists reported using therapeutic VR to treat people with chronic pain, and a regression analysis showed that the larger the physiotherapy practice, the more likely it was to use therapeutic VR [19]. Interestingly, our data also show that therapeutic VR was mainly used in larger facilities with more than 20 physiotherapists. It also emerged that physiotherapists in both the Netherlands and Germany use therapeutic VR with a wide range of age groups of patients and target coordination, mobility and pain relief for their patients. Similarly, the overall experience of therapeutic VR was comparable with 7.0 in the Netherlands and 6.43 in this study [19]. Looking at the reasons why physiotherapists who used to use therapeutic VR no longer do so, it is striking that in addition to cost, dissatisfaction with the results and negative patient experiences are the main reasons mentioned. This contrasts somewhat with reviews that report high levels of acceptability and feasibility of VR interventions [28]. However, there are also reviews that have looked at adverse events, such as cybersickness, which show that such events are still inaccurately and incompletely recorded and reported in therapeutic VR research [29].

Very little is known about the extent to which digital applications are used in physiotherapy in Germany. According to a survey conducted in 2018 and 2019, only 2.8% of physiotherapists use telemedicine and only 15% use electronic patient files [30]. However, much has changed in the area of digitalization since then, due to the COVID19 pandemic, and some implementation processes may have been accelerated, so the number is likely to be higher. Another indicator that usage could increase in the coming years is the positive attitude of physiotherapists and patients towards digital applications [31,32], as shown in other research that healthcare professionals play a key role in the implementation of digital interventions [33].

This study shows that many physiotherapists are also considering the use of therapeutic VR (76.7%). Focus groups with physiotherapists showed that they see great potential in therapeutic VR to open up new opportunities in rehabilitation [34]. However, there were still problems with implementation, such as the lack of evidence on the dosage of therapeutic VR [35]. VR devices themselves also appear to be a major barrier due to technological limitations that can make VR devices inappropriate for certain patients and their needs, such as those with neck pain. The software used can also be a barrier due to a lack of tutorials [16]. The main barrier in our survey seems to be that many have not received any information about the possibility of treatment with therapeutic VR (67.2%). This is in line with the results of the survey in the Netherlands, where 71.4% of physiotherapists reported that they were unfamiliar with therapeutic VR [19]. This barrier is also evident in the adoption of other digital health applications (Digitale Gesundheitsanwendungen [DiGA] [36]) in Germany. A survey showed that little, bad or no experience with applications is a barrier and that this leads to low abilities and confidence in using technology [37]. Therefore, part of a future strategy to increase the use of therapeutic VR and other digital applications could be to promote them to relevant professional groups through various channels. Healthcare professional education offers an important opportunity here, but it was found that digital health competencies are still underrepresented in physiotherapy education [35].

Only around 10% of physiotherapists say that costs are a barrier to use therapeutic VR in their treatment. However, it is still a problem that treatment with therapeutic VR or other digital applications cannot yet be reimbursed separately by health insurance companies. This means that any additional costs cannot be reimbursed by physiotherapists. The exception is digital health applications, but this register does not yet include any therapeutic VR [38].

Based on our results, future steps could be to taken to make therapeutic VR more widely known within the physiotherapy community, for example, through articles in practitioner journals or presentations at congresses. Another option would be to develop specific implementation strategies to support adoption and help ensure that therapeutic VR is used sustainably. Another possible step towards a more impactful use of VR would be a more precise understanding of how therapeutic VR works, for whom and at what dosage, in order to be able to make precise recommendations to physiotherapists.

## 5. Limitations

The survey also has various limitations. In regard to the sample, the number of participating physiotherapists did not reach the intended sample size of 384. At the same time, the sample of physiotherapists using VR shows slightly different distributions in terms of gender and academic degree compared to the total population. This can lead to bias, which may limit the generalization of the results. Similarly, the small number of physiotherapists who have used therapeutic VR in the last year means that the validity of our results is compromised in regard to factors that influence its use and in which areas therapeutic VR is primarily used.

## 6. Conclusions

This study provides a first insight into the adoption of therapeutic virtual reality (VR) in physiotherapy in Germany. Despite the potential of VR to improve healthcare treatments, its use among German physiotherapists remains limited, with only 2.7% having used it in the past year. The main barrier identified in this study is a lack of awareness, with 67.2% of non-users having never heard of therapeutic VR. Encouragingly, a significant proportion of physiotherapists are open to implement VR into their practice in the future. To facilitate wider adoption, focused efforts should be made to increase awareness and understanding of the benefits of VR within the physiotherapy community, supported by appropriate reimbursement strategies. Further research and targeted implementation strategies could promote the sustainable use of therapeutic VR in physiotherapy practices in Germany.

## Figures and Tables

**Table 1:** Demographic characteristics of participating physiotherapists.

	Physiotherapists Using VR	Physiotherapists Not Using VR	Total Sample
**N (%)**	8 (2.7)	288 (97.3)	296 (100)
**Age, median (IQ)**	47 (36)	46 (24)	46 (25)
**Gender, n (%)**			
Female	4 (50)	192 (66.7)	196 (66.2)
Male	3 (37.5)	93 (32.3)	96 (32.4)
Divers	1 (12.5)	1 (0.3)	2 (0.7)
No information	0	2 (0.7)	2 (0.7)
**Highest Degree, n (%)**			
Vocational certificate	1 (12.5)	201 (69.8)	202 (68.2)
Bachelor’s	4 (50)	53 (18.4)	57 (19.3)
Master’s	1 (12.5)	12 (4.2)	13 (4.4)
Doctoral	1 (12.5)	2 (0.7)	3 (1.0)
Other	1 (12.5)	14 (4.9)	15 (5.1)
No information	0	6 (2.1)	6 (2.0)
**Professional experience, n (%)**			
0–4 years	2 (25.0)	41 (14.2)	43 (14.5)
5–9 years	3 (37.5)	30 (10.4)	33 (11.1)
10–14 years	0	32 (11.1)	32 (10.8)
15–19 years	0	33 (11.5)	33 (11.1)
20+ years	2 (25.0)	145 (50.3)	147 (49.7)
No information	1 (12.5)	7 (2.4)	8 (2.7)
**Physiotherapists employed at the facility, n (%)**			
1	2 (25.0)	29 (10.1)	31 (10.5)
2–4	0	75 (26.0)	75 (25.3)
5–9	0	72 (25.0)	72 24.3
10–14	0	35 (12.2)	35 (11.8)
15–19	0	19 (6.6)	19 (6.4)
20–24	1 (12.5)	6 (2.1)	7 (2.4)
25–30	1 (12.5)	8 (2.8)	9 (3.0)
30+	4 (50.0)	39 (13.5)	43 (14.5)
No information	0	5 (1.7)	5 (1.7)
**Setting, n (%) ^1^**			
Outpatient practice	3 (37.5)	204 (70.8)	207 (69.9)
Acute care clinic	5 (62.5)	52 (18.1)	57 (19.3)
Inpatient rehabilitation	2 (25.0)	26 (9.0)	28 (9.5)
Outpatient rehabilitation	1 (12.5)	5 (1.7)	6 (2.0)
Other	1 (12.5)	19 (6.6)	19 (6.4)
**Specialization, n (%) ^1^**			
Musculoskeletal physiotherapy (orthopedics/manual therapy)	4 (50)	197 (68.4)	201 (67.9)
Post-operative rehabilitation	3 (37.5)	114 (39.6)	117 (39.5)
Pediatric physiotherapy	0	43 (14.9)	43 (14.5)
Neurological physiotherapy	5 (62.5)	114 (39.6)	119 (40.2)
Sports physiotherapy	1 (12.5)	55 (19.1)	56 (18.9)
Psychosomatic physiotherapy	2 (25.0)	19 (6.6)	21 (7.1)
Geriatric physiotherapy	2 (25.0)	63 (21.9)	65 (22.0)
Palliative care	0	27 (9.4)	27 (9.1)
Gynecology	1 (12.5)	32 (11.1)	33 (11.1)
No specialization	0	15 (5.2)	15 (5.1)
Other	0	30 (10.4)	30 (10.1)

^1^ More than one option possible.

**Table 2:** Characteristics of therapeutic VR use (n = 8).

Age of Patient Receiving Therapeutic VR, n (%) ^1^	
<18 years	3 (37.5)
18–30 years	2 (25)
31–50 years	4 (50)
51–70 years	4 (50)
>71 years	1 (12.5)
**Patient’s condition receiving therapeutic VR, n (%) ^1^**	
Neurological condition	6 (75)
Musculoskeletal condition	4 (50)
Chronic pain	4 (50)
Medically unexplained symptoms	3 (37.5)
Geriatrics	3 (37.5)
Pediatrics	1 (12.5)
Cardiopulmonary condition	1 (12.5)
Oncological condition	0
Other	0
No information	0
**Proposed working mechanism VR, n (%) ^1^**	
Activation	7 (87.5)
Relaxation	5 (62.5)
Reducing fear of movement	4 (50)
Education	3 (37.5)
Other	0
**Treatment goal of therapeutic VR, n (%) ^1^**	
Improve coordination	6 (75)
Improve physical mobility	5 (62.5)
Improve strength	5 (62.5)
Improve stability	4 (50)
Reduce pain	4 (50)
Improve endurance	2 (25)
Other	2 (25)
**Overall experience with therapeutic VR**	
0 (extremely bad) to 10 (extremely good)	6.43

^1^ More than one option possible.

**Table 3:** Previous use of therapeutic VR by physiotherapists (n = 17).

Reasons for Quitting Therapeutic VR, n (%) ^1^	
Costs	7 (41.2)
Dissatisfied with the results	5 (29.4)
Other reasons	5 (29.4)
Negative experiences of patients	2 (11.8)
Negative experiences of therapists	0
No information	4 (23.5)
**Patient’s condition receiving therapeutic VR, n (%) ^1^**	
Neurological condition	4 (23.5)
Musculoskeletal condition	10 (58.8)
Chronic pain	4 (23.5)
Medically unexplained symptoms	1 (5.9)
Geriatrics	0
Pediatrics	0
Cardiopulmonary condition	2 (11.8)
Oncological condition	0
Other	0
No information	2 (11.8)
**Consideration of future use of therapeutic VR, n (%)**	
Yes	10 (58.8)
No	3 (17.7)
Maybe	3 (17.7)
No information	1 (5.9)

^1^ More than one option possible.

**Table 4:** Future use of therapeutic VR by physiotherapists who have never used it before (n = 241).

Reasons Why Therapeutic VR Has Not Yet Been Used, n (%) ^1^	
I have never heard anything about it before	162 (67.2)
Other reasons	43 (17.8)
I don’t treat suitable patients	34 (14.1)
Costs	26 (10.8)
No information	11 (4.6)
**What conditions could you imagine using therapeutic VR for? n, (%) ^1^**	
Neurological condition	30 (12.4)
Musculoskeletal condition	101 (41.9)
Chronic pain	94 (39)
Medically unexplained symptoms	35 (14.5)
Geriatrics	50 (20.7)
Pediatrics	46 (19.1)
Cardiopulmonary condition	30 (12.4)
Oncological condition	30 (12.4)
Other	11 (4.6)
No information	82 (34)
**Consideration of future use of therapeutic VR, n (%)**	
Yes	83 (34.4)
No	44 (18.3)
Maybe	100 (41.4)
No information	14 (5.8)

^1^ More than one option possible.

**Table 5:** Comparison of the use of therapeutic VR by physiotherapists between Germany and the Netherlands [19].

	Physiotherapists Using VR	Physiotherapists Not Using VR	Total
**Germany,** n (%)	8 (2.7)	288 (97.3)	296
**Netherlands,** n (%)	18 (7.3)	227 (92.7)	345
**Total,** n (%)	26 (5.0)	515 (95)	541

## Data Availability

The data sets generated and analyzed during this study are available from the corresponding author on reasonable request.

## Appendix A. Survey

Questions 1–5: demographic characteristics of all physiotherapists

Questions 6–19: physiotherapists who currently use therapeutic VR

Questions 20–27: Physiotherapists who do not currently use therapeutic VR

1. To which gender do you assign yourself?
  - Male
  - Female
  - Diverse
2. How many employees does the physiotherapy practice where you work have?
  - 1
  - 2–4
  - 5–9
  - 10–14
  - 15–19
  - 20–24
  - 25–30
  - Over 30
3. How long have you been working as a physiotherapist?
  - 0–4 years
  - 5–9 years
  - 10–14 years
  - 15–19 years
  - 20 years or more
4. How old are you?
5. Have you specialized as a physiotherapist? *
  - No
  - Yes, in pediatrics
  - Yes, in neurology
  - Yes, in musculoskeletal physiotherapy (orthopaedics/manual therapy)
  - Yes, in sports physiotherapy
  - Yes, in psychosomatic physiotherapy
  - Yes, in gynecology
  - Yes, in geriatrics
  - Yes, in another area: (open question)
6. Have you used therapeutic virtual reality in the last year?
  - Yes
  - No
7. What is your general experience with therapeutic VR in your physiotherapy treatment?
  (Scale from 0 (very poor) to 10 (very good))
8. To what extent would you recommend therapeutic VR to a physiotherapist colleague (scale from 0 (I would definitely not recommend) to 10 (I would definitely recommend))?
9. How long have you been using therapeutic VR?
  - <1 year
  - 1–2 years
  - 3–5 years
  - >5 years
10. What age groups do you use therapeutic VR for?
  - <18 years old
  - 18–30 years old
  - 31–50 years old
  - 51–70 years old
  - 71 years old
11. For what conditions do you use therapeutic VR?
  - Musculoskeletal disorders
  - Medically unexplained physical symptoms
  - Heart, arterial or pulmonary disease
  - Neurological disorders
  - Geriatric diseases
  - Oncology
  - Pediatric Diseases
  - Other
12. For which musculoskeletal disorders do you use therapeutic VR?
  - Non-specific complaints of the cervical spine
  - Non-specific back pain
  - Arthritis
  - Headaches or dizziness
  - Pelvic or hip problems
  - Shoulder or arm pain
  - Fibromyalgia
  - Generalized pain complaints
  - Other
13. How many patients with chronic musculoskeletal pain do you treat with therapeutic VR as a percentage of your total patient population?
  - 0–10%
  - 11–25%
  - 26–40%
  - 41–60%
  - 61–75%
  - 76–80%
  - Above 80%
14. How do you use therapeutic VR?
  - Only in practice
  - Only in the patient’s home
  - Both
15. For which possible mechanism(s) of action do you use therapeutic VR? *
  - Education
  - Exposure therapy
  - Relaxation
  - Activation
  - Other
16. For which treatment goals do you use therapeutic VR? *
  - Improving physical mobility
  - Improving stability
  - Improve strength
  - Reduce pain
  - Improve Endurance
  - Improve coordination
  - Other
17. What kind of hardware are you using for therapeutic VR? *
  - VR headset
  - Nintendo Wii
  - Xbox Kinect
  - Other
18. What type of VR headset do you use for therapeutic VR? *
  - Oculus Go
  - Oculus Quest
  - Oculus Rift (S)
  - Samsung Gear
  - HTC Vive
  - Valve Index
  - Vive Force
  - Pico G2
  - Pico Neo 3
  - Pico 4
  - Other
19. What type of therapeutic VR software do you use? *
  - Reducept
  - SyncVR Fit
  - VRelax
  - Corpus VR (InMotion)
  - VRendle
  - Kana
  - SyncVR Relax & Distract
  - Not applicable
  - Other

From here for those who are not currently using it:

20. Have you used therapeutic VR in the past?
  - Yes
  - Never
21. Why have you stopped using therapeutic VR? *
  - Cost
  - Dissatisfied with the results
  - Negative patient experience
  - Negative experiences with physical therapists
  - Other reasons
22. Would you consider using therapeutic VR again?
  - I would
  - Would not
23. Why do you not use therapeutic VR? *
  - Cost
  - I am not treating appropriate patients
  - I have never read about it
  - Other reasons
24. For which conditions would you like to use therapeutic VR? *
  - Musculoskeletal disorders
  - Medically unexplained physical symptoms
  - Heart, arterial or pulmonary disease
  - Neurological disorders
  - Geriatric diseases
  - Oncology
  - Pediatric Diseases
  - Other
25. For which potential mechanism(s) of action would you like to use therapeutic VR? *
  - Education
  - Exposure therapy
  - Relaxation
  - Activation
  - Other
26. For which treatment goals would you like to use therapeutic VR? *
  - Improve physical mobility
  - Improve stability
  - Improve strength
  - Reduce pain
  - Improve Endurance
  - Improve coordination
  - Other
27. Why do you not use therapeutic VR? *
  - Cost
  - I do not have suitable patients
  - I have never read about it
  - Other reasons

* More than one option possible
